# Barriers and Facilitators to Clinician Readiness to Provide Emergency Department–Initiated Buprenorphine

**DOI:** 10.1001/jamanetworkopen.2020.4561

**Published:** 2020-05-11

**Authors:** Kathryn F. Hawk, Gail D’Onofrio, Marek C. Chawarski, Patrick G. O’Connor, Ethan Cowan, Michael S. Lyons, Lynne Richardson, Richard E. Rothman, Lauren K. Whiteside, Patricia H. Owens, Shara H. Martel, Edouard Coupet, Michael Pantalon, Leslie Curry, David A. Fiellin, E. Jennifer Edelman

**Affiliations:** 1 Department of Emergency Medicine, Yale School of Medicine, New Haven, Connecticut; 2 Department of Psychiatry, Yale School of Medicine, New Haven, Connecticut; 3 Department of Internal Medicine, Yale School of Medicine, New Haven, Connecticut; 4 Department of Emergency Medicine, Icahn School of Medicine at Mount Sinai, New York, New York; 5 Department of Emergency Medicine, University of Cincinnati, Cincinnati, Ohio; 6 Department of Emergency Medicine, Johns Hopkins University School of Medicine, Baltimore, Maryland; 7 Department of Emergency Medicine, University of Washington, Seattle; 8 Department of Health Policy and Management, Yale School of Public Health, New Haven, Connecticut

## Abstract

**Importance:**

Treatment of opioid use disorder (OUD) with buprenorphine decreases opioid use and prevents morbidity and mortality. Emergency departments (EDs) are an important setting for buprenorphine initiation for patients with untreated OUD; however, readiness varies among ED clinicians.

**Objective:**

To characterize barriers and facilitators of readiness to initiate buprenorphine for the treatment of OUD in the ED and identify opportunities to promote readiness across multiple clinician types.

**Design, Setting, and Participants:**

Using data collected from April 1, 2018, to January 11, 2019, this mixed-methods formative evaluation grounded in the Promoting Action on Research Implementation in Health Services framework included 4 geographically diverse academic EDs. Attending physicians (n = 113), residents (n = 107), and advanced practice clinicians (APCs) (n = 48) completed surveys electronically distributed to all ED clinicians (n = 396). A subset of participants (n = 74) also participated in 1 of 11 focus group discussions. Data were analyzed from June 1, 2018, to February 22, 2020.

**Main Outcomes and Measures:**

Clinician readiness to initiate buprenorphine and provide referral for ongoing treatment for patients with OUD treated in the ED was assessed using a visual analog scale. Responders (268 of 396 [67.7%]) were dichotomized as less ready (scores 0-6) or most ready (scores 7-10). An ED-adapted Organizational Readiness to Change Assessment (ORCA) and 11 focus groups were used to assess ratings and perspectives on evidence and context-related factors to promote ED-initiated buprenorphine with referral for ongoing treatment, respectively.

**Results:**

Among the 268 survey respondents (153 of 260 were men [58.8%], with a mean [SD] of 7.1 [9.8] years since completing formal training), 56 (20.9%) indicated readiness to initiate buprenorphine for ED patients with OUD. Nine of 258 (3.5%) reported Drug Addiction Treatment Act of 2000 training completion. Compared with those who were less ready, clinicians who were most ready to initiate buprenorphine had higher mean scores across all ORCA Evidence subscales (3.50 [95% CI, 3.35-3.65] to 4.33 [95% CI, 4.13-4.53] vs 3.11 [95% CI, 3.03-3.20] to 3.60 [95% CI, 3.49-3.70]; *P* < .001) and on the Slack Resources of the ORCA Context subscales (3.32 [95% CI, 3.08-3.55] vs 3.0 [95% CI, 2.87-3.12]; *P* = .02). Barriers to ED-initiated buprenorphine included lack of training and experience in treating OUD with buprenorphine, concerns about ability to link to ongoing care, and competing needs and priorities for ED time and resources. Facilitators to ED-initiated buprenorphine included receiving education and training, development of local departmental protocols, and receiving feedback on patient experiences and gaps in quality of care.

**Conclusions and Relevance:**

Only a few ED clinicians had a high level of readiness to initiate buprenorphine; however, many expressed a willingness to learn with sufficient supports. Efforts to promote adoption of ED-initiated buprenorphine will require clinician and system-level changes.

## Key Points

**Question:**

What are the key barriers and facilitators for clinician initiation of buprenorphine in the ED with referral for the treatment of opioid use disorder?

**Findings:**

A mixed-methods evaluation of 268 attending physicians, resident physicians, and advanced practice clinicians in academic emergency departments found that 9 of 258 (3.5%) reported completion of Drug Addiction Treatment Act of 2000 training, and 56 of 268 (20.9%) had high levels of readiness to prescribe buprenorphine in the emergency department. Key barriers included lack of training and experience in treating opioid use disorder, concerns about ability to link to ongoing care, and competing needs for time and resources in a busy emergency department.

**Meaning:**

Although few emergency department clinicians in this study had high levels of readiness to initiate buprenorphine with referral for ongoing treatment, many expressed a willingness to learn with sufficient support, indicating the importance of clinician and system-level changes.

## Teaser

This qualitative analysis surveys barriers and facilitators of readiness to initiate buprenorphine administration in the emergency department and identifies opportunities to promote readiness among clinicians to treat patients with opioid use disorder.

## Introduction

In 2018, an estimated 2.0 million individuals in the United States had an opioid use disorder (OUD).^1,2^ Treatment with methadone or buprenorphine can mitigate adverse consequences and decrease mortality.^3,4,5,6,7^ Despite the strong evidence and clinical guidelines supporting use of these medications,^8,9,10^ as many as 80% of patients do not receive such potentially life-saving treatments.^7,11^

To address this treatment gap, the emergency department (ED) has emerged as an important location to identify patients with OUD and initiate buprenorphine with referral for ongoing treatment.^12^ Buprenorphine is a safe, effective OUD treatment that rapidly relieves withdrawal, may be dispensed by any licensed clinician in an ED, and unlike methadone, may be prescribed to outpatients for the treatment of OUD by any clinician who has completed approved training and received a Drug Addiction Treatment Act of 2000 (DATA 2000) waiver.^8,13^ Furthermore, previous studies^14,15^ demonstrated that when patients with OUD receive ED-initiated buprenorphine treatment with referral to ongoing care, the process promotes addiction treatment engagement and is cost-effective. Despite these data, adoption of ED-initiated buprenorphine nationwide has been limited.^11,16,17^ Thus, to inform future implementation efforts to promote ED-initiated buprenorphine with referral for ongoing treatment, we conducted a mixed-methods formative evaluation involving attending physicians, resident physicians, and advanced practice clinicians (APCs), including physician assistants and nurse practitioners, in 4 geographically disparate EDs across the United States.

## Methods

### Overview of Project ED Health

A mixed-methods formative evaluation was conducted in the context of the ongoing Project ED Health,^18,19^ a study funded by the National Institute on Drug Abuse Center for the Clinical Trials Network in 4 geographically diverse EDs through January 2021. The study, a hybrid type III effectiveness-implementation trial,^20^ was designed to evaluate the effect of implementation facilitation^21^ vs a standard educational dissemination strategy on ED-initiated buprenorphine treatment.^18^ The present study was approved by the Western Institutional Review Board. The participants in this baseline evaluation of Project ED Health provided verbal informed consent.

The study approach was guided by the Promoting Action on Research Implementation in Health Services (PARiHS) framework. This framework is often used to understand how the interplay between perspectives on the evidence of the target evidence-based practice interacts with the context for delivering that practice and the facilitation strategy.^22,23,24^ Our quantitative approach was consistent with the American Association for Public Opinion Research guidelines as outlined in the Internet Surveys of Specifically Named Persons,^25^ and our qualitative approach adhered to the Consolidated Criteria for Reporting Qualitative Research (COREQ) reporting guideline.^26^

### Setting and Participants

Project ED Health is being conducted in 4 academic, urban EDs in Baltimore, Maryland (annual visit volume, >70 000), New York, New York (annual visit volume, >90 000), Cincinnati, Ohio (annual visit volume, >75 000), and Seattle, Washington (annual visit volume, >60 000).^18^ Clinicians who were potential ED-based buprenorphine prescribers, including attending physicians, residents, and APCs (inclusive of physician assistants and nurse practitioners), and who had been employed at the respective EDs for at least 6 months before the study onset (n = 396) were invited to participate. Of those, 268 (67.7%) provided a response to the readiness question included in the online survey. Among the 396 clinicians invited to take the survey, 74 prescribers, including 37 attending physicians, 25 residents, and 12 APCs, participated in 4 faculty, 4 resident, and 3 APC focus groups.^18^ All focus group participants provided verbal informed consent.

### Data Collection

The mixed-methods formative evaluation included quantitative and qualitative assessments informed by the PARiHS framework.^23,27^ Data collection for the present analysis occurred from April 1, 2018, through January 11, 2019, after a single Grand Rounds on ED-initiated buprenorphine by a lead study investigator (G.D.) and before any further study-related implementation efforts.

#### Quantitative

 The quantitative assessment included a web-based, anonymous survey that collected self-reported data on demographic characteristics, training, and experiences with ED-initiated buprenorphine; ratings on the Evidence and Context subscales of the ED-adapted Organizational Readiness to Change Assessment (ORCA) (eMethods in the Supplement); and a visual analog scale using a readiness ruler that asked: “On a scale from 0 to 10, how ready are you to provide ED-initiated buprenorphine with referral for ongoing MAT [medication for addiction treatment] for the treatment of opioid use disorder, where 0 equals ‘not ready at all’ and 10 equals ‘totally ready?’”^28^

The ED-adapted ORCA assesses perspectives on evidence and context for ED-initiated buprenorphine with referral for ongoing treatment.^18^ The Evidence scale includes 4 subscales: Staff Discord (ie, perspectives on evidence among the respondent and their colleagues), Research Evidence, Clinical Practice Experience, and Patient Needs.^29^ Responses were collected on a 5-point Likert scale (1 indicates strongly disagree; 2, disagree; 3, neither agree nor disagree; 4, agree; and 5, strongly agree). The Context scale contains 5 subscales: Leadership Culture, Staff Culture (ie, staff organizational culture), Leadership Practice, Evaluation Accountability, and Opinion Leader Culture. An additional item assesses resources available to support practice change (Slack Resources).^30^ Responses were collected on a 5-point Likert scale (1 indicates very infrequently; 2, infrequently; 3, neither frequently nor infrequently; 4, frequently; and 5, very frequently). Answer choices “don’t know” and “not applicable” were also included for both scales. Subsequently, responses “don’t know” and “not applicable” were recoded as neither agree nor disagree or neither frequently nor infrequently, and subscale scores for the Evidence and Context domains were computed.

#### Qualitative

Qualitative data collection occurred through focus groups in a nonclinical space, with an a priori plan to have 1 faculty, resident, and APC focus group per site consistent with the number of focus groups typically required to achieve thematic saturation.^31^ Participants were volunteers invited through in-person and electronic communications. Refusals were not tracked. No participant dropped out during the focus group meetings. The focus group facilitators included authors (K.F.H., G.D., P.G.O., D.A.F., and E.J.E.) who identified themselves to the group as physician researchers trained in addiction medicine, emergency medicine, and/or internal medicine. A previously published focus group guide included grand tour questions with follow-up probes designed to elicit perspectives on prior experiences with treating OUD; understanding of the evidence supporting use of ED-initiated buprenorphine with referral for ongoing treatment; contextual factors related to ED-initiated buprenorphine with referral for ongoing treatment, including administrative and leadership support; and resources needed to implement this practice.^18,32^ All focus groups were digitally recorded and transcribed.

### Data Analyses

#### Quantitative

Survey data were analyzed from March 7, 2019, to February 22, 2020. Descriptive statistics were used to characterize participant characteristics. After a review of the distributional properties of responses to the readiness ruler question, participants in the bottom 4 quintiles (scores 0-6) were categorized as less ready, and participants in the top quintile (scores 7-10) were categorized as most ready. The 2 groups were then compared on their characteristics, their DATA 2000 waiver training status, and the ORCA subscales in an exploratory fashion. No a priori hypotheses were formulated. The statistical significance of differences between the 2 readiness level groups on their characteristics and the DATA 2000 waiver training status were evaluated using the Pearson χ^2^ test or the Fisher exact test (when the cell counts were less than 5). The difference in the years since completing clinical training was evaluated using the independent samples *t* test. The between-group differences on the ORCA subscales were evaluated using a multivariate analysis of variance with the readiness level (less ready vs most ready) as the between-group factor and including the site and clinician type (attending, resident, or APC) as covariate terms in the general linear multivariate model. The significance levels of the observed between-group differences were assessed using the F test statistic. A sensitivity analysis to compare the patterns of between-group differences on the ORCA subscales where the “don’t know” and “not applicable” responses were excluded and where they were recoded as neither agree nor disagree or neither frequently nor infrequently before computing the scores on the ORCA subscales was also conducted. Statistical analyses were performed using SPSS, version 24 (IBM Corporation). Two-tailed *P* < .05 indicated statistical significance.

#### Qualitative

Informed by the PARiHS framework, we used an inductive process of iterative coding to identify ideas and themes that was interpolated with conduction of focus groups.^33^ Three members of the investigative team (K.F.H., E.C., Jr, and E.J.E.) independently reviewed 7 transcripts line by line to develop and refine the codebook and reach consensus on codes and reached thematic saturation.^31^ An audit trail was maintained. One investigator (E.J.E.) then applied these codes consistently to all transcripts, using Atlas.ti software (ATLAS.ti Scientific Software Development) to facilitate data organization and retrieval. Themes were then generated based on coded quotations through discussion with the research team within the PARiHS framework.

#### Data Integration

Data were collected using a sequential explanatory strategy,^34^ with quantitative data collection preceding focus groups at each site. We used qualitative data to contextualize quantitative findings and triangulate the collected data.^20^

## Results

### Participant Demographic and Professional Characteristics

Of the 268 of 396 clinicians (67.7%) who completed the readiness ruler, 113 (42.2%) were attending physicians, 107 (39.9%) were residents, and 48 (17.9%) were APCs (Table 1). The majority of participants were male (153 of 260 were men [58.8%] and 107 of 260 were women [41.2%]) and white (205 of 240 [85.4%]). Nine of 258 participants (3.5%) reported having completed DATA 2000 training, including 5 attending physicians, 3 residents, and 1 APC. High readiness to initiate buprenorphine in the ED was endorsed by 56 participants overall (20.9%), including 24 of 113 attending physicians (21.2%), 26 of 107 residents (24.3%), and 6 of 48 APCs (12.5%).

**Table 1.** Participant Characteristics, Overall and by Readiness to Prescribe ED-Initiated Buprenorphine With Referral for Ongoing Treatment

Characteristic	Clinician group		*P* value for less vs most ready	Overall, No./total No. (%)
	Less ready	Most ready		
Sex, No./total No. (%)				
Male	119/207 (57)	34/53 (64)	.38^a^	153/260 (58.8)
Female	88/207 (43)	19/53 (36)		107/260 (41.2)
No./total No. (%) white	165/193 (85)	40/47 (85)	>.99^a^	205/240 (85.4)
Clinician type, No./total No. (%)				
Attending	89/212 (42)	24/56 (43)	.25^a^	113/268 (42.1)
Resident	81/212 (38)	26/56 (46)		107/268 (39.9)
APC	42/212 (20)	6/56 (11)		48/268 (17.9)
DATA 2000 training, No/total No. (%)	4/204 (2.0)	5/54 (9.3)	.02^b^	9/258 (3.5)
Time since completing clinical training, mean (SD), y	7.14 (9.24)	6.82 (11.74)	.98^c^	7.07 (9.79)

Abbreviations: APC, advanced practice clinicians; DATA 2000, Drug Addiction Treatment Act of 2000; ED, emergency department.

^a^ Compared using the Pearson χ^2^ test.

^b^ Compared using the Fisher exact test.

^c^ Compared using the independent-samples *t* test.

### Factors Associated With Readiness to Provide ED-Initiated Buprenorphine

**Figure 1.**
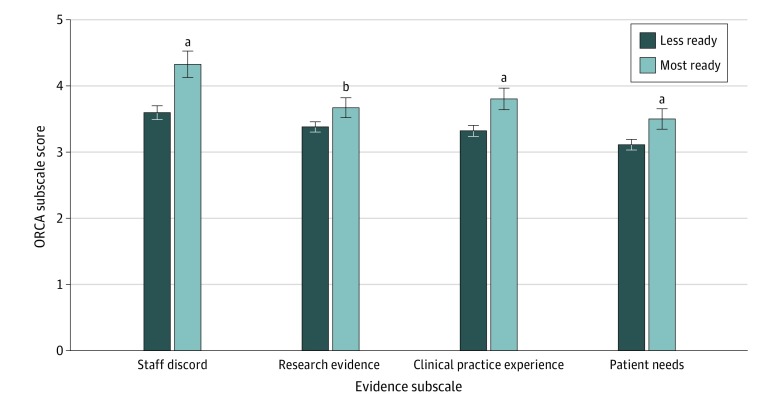
Organizational Readiness to Change Assessment (ORCA) Evidence Subscales by Readiness to Initiate Buprenorphine Treatment in the Emergency Department Data are expressed as mean scores; error bars indicate 95% CI. ^a^*P* < .001 ^b^*P* = .001

**Figure 2.**
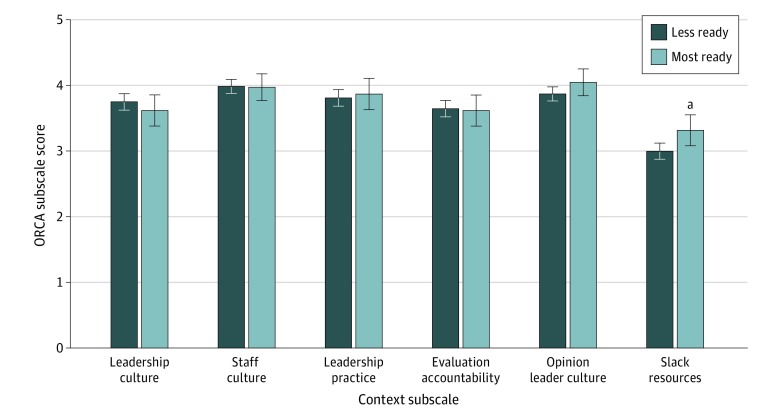
Organizational Readiness to Change Assessment (ORCA) Context Subscales by Readiness to Initiate Buprenorphine Treatment in the Emergency Department Data are expressed as mean scores; error bars indicate 95% CI. ^a^*P* = .02.

Completion of DATA 2000 training was associated with higher levels of readiness for ED-initiated buprenorphine prescribing with referral for ongoing treatment (5 of 9 [55.6%]) compared with no DATA 2000 training (49 of 249 [19.7%]; χ^2^ = 6.756; *P* = .02). In addition, clinicians with higher levels of readiness to initiate buprenorphine with referral for ongoing treatment had significantly higher mean scores on all ORCA Evidence subscales than clinicians with lower levels of readiness, including Staff Discord (4.33 [95% CI, 4.13- 4.53] vs 3.60 [95% CI, 3.49-3.70]; *P* < .001), Research Evidence (3.67 [95% CI, 3.52-3.82] vs 3.38 [95% CI, 3.30-3.46]; *P* = .001), Clinical Practice Experience (3.80 [95% CI, 3.64-3.97] vs 3.32 [95% CI, 3.24-3.40]; *P* < .001), and Patient Needs (3.50 [95% CI: 3.35-3.65] vs 3.11 [95% CI, 3.03-3.20]; *P* < .001) (Figure 1). In a sensitivity analysis, the overall pattern of the between-group differences on the means of all subscales as well as the pattern of the corresponding statistical significance levels closely overlapped with the notable change of the Patient Needs subscale that was no longer statistically significant. Those most ready compared with less ready had the following mean scores on the Context subscales: Leadership Culture (3.62 [95% CI, 3.38-3.85] vs 3.75 [95% CI, 3.62-3.87]; *P* = .34), Staff Culture (3.97 [95% CI, 3.77-4.12] vs 3.98 [95% CI, 3.88-4.09]; *P* = .91), Leadership Practice (3.87 [95% CI, 3.63-4.11] vs 3.81 [95% CI, 3.68-3.94]: *P* = .67), Evaluation Accountability (3.62 [95% CI, 3.38-3.85] vs 3.65 [95% CI, 3.52-3.77]; *P* = .83), Opinion Leader Culture (4.05 [95% CI, 3.84-4.25] vs 3.87 [95% CI, 3.76-3.98]; *P* = .13), and Slack Resources (3.32 [95% CI, 3.08-3.55] vs 3.0 [95% CI, 2.87-3.12]; *P* = .02) (Figure 2).

### Qualitative Themes

Themes with representative quotations are organized by PARiHS domains of Evidence and Context. Factors associated with readiness for ED-initiated buprenorphine treatment are identified in Table 2.

**Table 2.** Themes and Illustrative Quotations Organized by ORCA Subscale

ORCA subscale	Theme	Illustrative quote (clinician)
Evidence		
Staff Discord	Protocols mitigate variations in adoption of practice change, particularly within hierarchy	“If some of this can be evidence-based and we can cognitively offload, and we can have a system for doing this, that would be easier.” (attending)
		“We have the evidence to support, but also a very clear algorithm. I get a lot more acceptance to things that prior—before, that I would have deviate [my treatment plan] based on [which faculty member] I was talking to.” (resident)
Research Evidence	Vague knowledge of the research supporting ED-initiated buprenorphine treatment was common, but knowledge needed for implementation was limited	“Trying to suss out which of those patients might be appropriate for initiating some therapy and which aren’t is a skill that I don’t have. I don’t think that it’s a skill that we’re necessarily being trained for right now.” (resident)
		“I was there at the grand rounds that you came to. I remember a little.” (APC)
Clinical Practice Experience	ED clinicians report little formal training in addiction or the treatment of OUD, but view the waiver as a barrier	“That’s not something that we’re even really taught in medical school and certainly not in our training as emergency physicians. It is this detox black box across the street, and that’s how it is in many places.” (attending)
		“It’s another amount of paperwork and charting and licensure. Applying for a waiver now to do such a thing is another step.” (APC)
Patient Needs	Traditional ED care of patients with OUD is inadequate	“I feel like this is particularly vulnerable patient population that we’re just saying: ‘Here’s a sheet. Call some numbers. Good luck.’ That’s the way it feels when I discharge these folks.” (attending)
Context		
Leadership Culture and Practice	Leadership buy-in influences implementation	“We would probably look to our leadership if that was something, they would encourage us to do.” (APC)
Staff Culture	Make it easy/protocols drive care	“It’s all about the spin you put on it. Definitely, we don’t want somebody sitting in a room for an hour while we’re waiting for the pharmacist to be able to educate them and then doing the actual education and waiting for the suboxone to come out, but I think, if you could say, ‘This is going to be a quick thing. It may decrease future ED visits.’” (attending)
	Perceived scope of emergency medicine and competing demands on time	“As an emergency physician, that hasn’t been part of our culture to start any kind of long-term therapy.” (attending)
		“It’s a matter of limited resources in the emergency department. Every minute that I’m writing a suboxone prescription is a minute that I’m not with my critically ill patient.” (attending)
Evaluation Accountability	Concerns about long term follow-up	“I think just the resistance in, actually, prescribing it in the ED and making the referrals is just knowing exactly—how is the patient going be able to get there, and where, specifically, should I be sending them?” (attending)
Opinion Leader Culture	Local clinical expertise is valued and can drive practice change	“The [navigator] would come and talk to me about which interventions they thought each patient was ready for. That was a really valuable resource in terms of deciding how much effort to exert on each patient’s behalf.” (resident)
Slack Resources	Strong desire for staffing resources to support implementation	“We can’t provide all of that care up front. It’s just too time consuming and there are other patients to see. I think that would be one barrier. I think generally, yes, they would be supportive as long as they felt like they were supported by the institution and the resources available.” (resident)

Abbreviations: APC, advanced practice clinician; ED, emergency department; ORCA, Organizational Readiness to Change Assessment; OUD, opioid use disorder.

### Perspectives on Evidence for ED-Initiated Buprenorphine

Lack of training or personal experience initiating buprenorphine in the ED and absence of clear department protocols were identified as barriers to initiating buprenorphine with referral for treatment in the ED. Variability across clinician types in readiness to use buprenorphine was widespread. Key facilitators included widespread resident and APC enthusiasm to obtain training and implement practice change as well as a broad desire to improve outcomes for patients with OUD treated in the ED.

#### Staff Discord

Mixed levels of knowledge and awareness of evidence supporting the initiation of buprenorphine with referral for ongoing treatment in the ED were evident, as were differences in the readiness to implement practice change across prescriber type. Although several individual attending physicians expressed enthusiasm, residents and APCs were more enthusiastic about learning how to initiate buprenorphine in the ED, although they still highlighted the need for clear support from departmental leadership and the development of local protocols and clinical guidelines to facilitate their ability to implement practice change.

#### Research Evidence

With the exception of a few local champions, a vague understanding of the concept of ED-initiated buprenorphine was common. Some prescribers expressed concern that their patient population may be too medically or socially complex, but nearly all expressed a desire to learn more about the evidence supporting the use of buprenorphine, with the highest level of notable enthusiasm coming from residents and APCs.

#### Practice Experience and Patient Needs

Despite acknowledgment of a high prevalence of OUD among their patients, limited formal training on the treatment of OUD, including buprenorphine, was widely noted by participants. Specifically, participants believed themselves to be underprepared to identify patients appropriate for buprenorphine initiation and expressed lack of clear understanding of the regulations and laws guiding buprenorphine prescribing. Although the need for training was recognized, the requirement for DATA 2000 waiver training was perceived as a barrier. This contrasted with a recognition of the inadequacy of the traditional approach to OUD treatment with “handing out a pamphlet” and some early clinician experiences with dispensing buprenorphine in the ED to enhance overall readiness for adopting ED-initiated buprenorphine.

### Perspectives on Context for ED-Initiated Buprenorphine

Conflicting perceptions about the scope of emergency care and the perceived role of the ED in initiating treatment for OUD were recurrent contextual barriers to initiating buprenorphine in the ED. Key facilitators include leadership support, leveraging reliance on protocols to improve care, quality improvement with audit and feedback, and the availability of multidisciplinary support resources.

#### Leadership Culture and Practice

Across clinician types, the need for a commitment from departmental leadership to support practice change was recognized. Residents and APCs highlighted the importance of leadership support in the educational as well as administrative sphere as a key component needed to facilitate practice change. One perceived important effect of leadership commitment was the dedication of resources to develop and implement local protocols and care pathways to facilitate practice change; development and implementation of a clinical protocol was consistently perceived as a necessary step to facilitate adoption of ED-initiated buprenorphine. Clinicians at each site reported examples of other successfully implemented ED protocols with clinical decision support that yielded streamlined and standardized care across clinicians. Residents and APCs noted that the existence of protocols would serve as helpful leverage when working with attendings who had not yet integrated the use of buprenorphine into their practices.

#### Staff Culture

Clinicians varied in their perspectives on whether they believed that it was appropriate to initiate buprenorphine in the ED for patients with OUD as part of routine care. On one hand, some prescribers believed that it was more appropriate to prioritize care of patients with higher illness acuity over management of a chronic disease. On the other hand, some clinicians recognized that the ED offered an opportunity to initiate care that may not be provided otherwise. Similarly, although many clinicians were concerned about how much time initiating buprenorphine in the ED would take, others recognized that once fluent, this practice change may enhance ED flow, particularly if interdisciplinary support resources are used.

#### Evaluation Accountability

Uncertainty about whether the patient will be able to access and reliably attend follow-up appointments was one of the most commonly cited clinician concerns about initiating buprenorphine in the ED. Clinicians noted that receiving feedback about successful follow-up treatment of patients who experienced ED-initiated buprenorphine would enhance practice change and enhance readiness to initiate buprenorphine with referral for ongoing treatment in the ED.

#### Opinion Leader Culture

Although experience with initiating buprenorphine in the ED across sites was limited, many clinicians favorably described the presence of early adopters of ED-initiated buprenorphine in their EDs (primarily attending physicians) who were working to influence practice change. The presence of multiple hospital and city-wide task forces to actively support the expansion of access to OUD treatment was noted.

#### Slack Resources

A key contextual factor shared across sites and prescriber types was the identified need of multidisciplinary support to identify and educate patients and to facilitate follow-up logistics. Although specific requests varied among an addiction consultation team, peer navigators, nursing screening and assessments, or social work support, the request for support was universal.

## Discussion

To our knowledge, this study is the first to apply mixed methods to characterize factors associated with adoption of ED-initiated buprenorphine with referral for ongoing treatment from the perspectives of a diverse set of potential prescribers practicing in different regions of the United States. Our study reveals several key findings. Of 258 ED clinicians who responded, only 56 (20.9%) rated their readiness to initiate buprenorphine as 7 or more on a visual analog scale. Barriers to adoption of ED-initiated buprenorphine include a lack of formal training, limitations on time, limited knowledge of local treatment resources, absence of local protocols and referral networks, and perception or culture that this falls outside the scope and practice of emergency medicine. Facilitators include provision of education and training, establishment of protocols, and enhanced communication across different stakeholder groups. Together, these data are critical for guiding future implementation efforts to ensure that more patients with OUD receive potentially life-saving treatment with buprenorphine when seen in the ED.

Higher levels of readiness for prescribing buprenorphine in the ED were associated with higher scores on all ORCA Evidence subscales, whereas on the Context subscales, only higher Slack Resources subscale scores were associated with higher levels of readiness for ED-initiated buprenorphine with referral for ongoing treatment. As supported by the sensitivity analysis, the overall pattern of these between-group differences and the corresponding statistical significance levels were closely overlapping when the “don’t know” and “not applicable” ORCA responses were excluded and when they were recoded as neither agree nor disagree or neither frequently nor infrequently.

Despite strong endorsement of ED-initiated buprenorphine with referral for outpatient follow-up by the American College of Emergency Physicians, the American College of Medical Toxicology, and the National Institute on Drug Abuse, little has been published to date on ED clinician perspectives on initiating buprenorphine with referral for ongoing treatment in the ED.^10,13,14,16,35,36^ Consistent with our findings, 1 cross-sectional survey of 82 ED physicians within 1 health system also found that most participants were not prepared to initiate buprenorphine in the ED.^37^ A behavioral analysis approach to evaluating the effect of a campaign to increase DATA 2000 training completion among emergency physicians identified key barriers of absent social norms, the hassle of obtaining a waiver, and lack of salience in treating OUD in the ED.^17^ The need for a DATA 2000 waiver was identified as a barrier to initiating buprenorphine in the ED in our study as well, although there was some indication that an oversimplified view of the waiver as the main barrier may eclipse other areas of clinician resistance or factors slowing implementation by providing an easy answer for why clinicians do not view initiating buprenorphine as in their scope of practice.

Collectively, our study highlights several key strategies that may be useful for promoting adoption of ED-initiated buprenorphine. First, clinicians expressed the most interest in adopting practice change if they thought that it would improve patient outcomes. Second, we identified unique enthusiasm among residents and the potential for “teaching up,” in which practice change (eg, point of care ultrasonography) is driven by the delivery of new knowledge in medical and graduate curriculums, generating students, residents, and recent graduates with unique expertise to share with more established clinicians who want to keep up with evolving or novel clinical practices.^38^ Third, consistent with other ED-based quality improvement initiatives (care of sepsis and ST-elevation myocardial infarction), feedback on the number of patients with OUD who had treatment initiated in the ED and linked to ongoing care, coupled with individual success stories, could be powerful motivators to enhance practice change. Fourth, the importance of protocols cannot be overstated. They signify leadership and multistakeholder engagement and provide clear clinical evidence and guidelines about the goals of ED care of patients with OUD. Last, ED clinicians expressed a need for training, in addition to resources and personnel to support identification of patients who are appropriate for initiation of buprenorphine and to enhance treatment motivation and facilitate successful transitions of care.

### Limitations

Our study has some limitations. First, our findings may be subject to selection bias, recall bias, and social desirability bias owing to the conduct of the focus groups by study authors, including the previous grand rounds speaker. Two authors (K.F.H. and E.J.E.) facilitated focus groups and were part of the coding team, although potential effects of this dual role were mitigated by having each focus group include at least 1 facilitator not involved in the analysis and a member of the coding team (E.C. Jr) who did not conduct any focus groups. Across sites, research assistants purposively recruited participants based on clinical scheduling and existing meetings unrelated to our study to include a variety of perspectives on ED-initiated buprenorphine rather than those self-selecting to participate. Survey participation was deidentified, with some clinical and professional characteristics but no names, so direct correlation of survey data with focus group data was not possible. Second, our findings may not be generalizable to rural or community-based EDs. Third, perspectives and practices are rapidly evolving across these EDs, given the ongoing opioid epidemic, whereas the study findings are based on assessments at 1 time per site.

## Conclusions

This study’s findings suggest that opportunities to promote readiness for ED-initiated buprenorphine with referral for ongoing treatment will need to address perspectives on the evidence and ED context for implementation. Specific opportunities to promote practice change vary across emergency clinician type. Future implementation strategies should consider these factors and tailor interventions accordingly for attending physicians, residents, and APCs.

## Notes

Supplement. **eMethods.** Implementation Facilitation–Formative Evaluation
