# Supplementary material for: Barriers and Facilitators to Clinician Readiness to Provide Emergency Department–Initiated Buprenorphine
Source: JAMA Netw Open. 2020 May 11;3(5):e204561. doi: 10.1001/jamanetworkopen.2020.4561 (PMC7215257; doi:10.1001/jamanetworkopen.2020.4561)
Supplement: Supplement. — eMethods. Implementation Facilitation–Formative Evaluation [file jamanetwopen-3-e204561-s001.pdf]

Supplementary Online Content

Hawk KF, D'Onofrio G, Chawarski MC, et al. Barriers and facilitators to clinician readiness to provide emergency department–initiated buprenorphine. *JAMA Netw Open*. 2020;3(5):e204561. doi:10.1001/jamanetworkopen.2020.4561

eMethods. Implementation Facilitation–Formative Evaluation

This supplementary material has been provided by the authors to give readers additional information about their work.

©2020 Hawk KF et al. *JAMA Network Open.*

eMethods. Implementation Facilitation–Formative Evaluation

Organizational Readiness to Change Assessment for ED Providers/Staff (ORCA)

We need your help assessing your and your colleagues' readiness to implement ED-initiated buprenorphine with referral for ongoing medication assisted treatment (MAT) for opioid use disorder in your Emergency Department (ED). By opioid use disorder we mean patients with uncontrolled use (addiction) of illicit (e.g. heroin) or prescription opioids. By MAT, we mean use of medications for the treatment of opioid use disorder. (i.e. buprenorphine, methadone, naltrexone). By treatment engagement, we mean that the patient is receiving treatment for their opioid use disorder with MAT. In the Evidence section below, we ask you to provide your opinions regarding buprenorphine. In the Context section that follows, we ask some questions about features of your ED. Please consider each question carefully and select the answer that best reflects your opinion.

I. EVIDENCE ASSESSMENT FOR EACH INTERVENTION

The following set of questions are about the evidence that ED-initiated buprenorphine with referral for ongoing medication assisted treatment (MAT) to community-based practices/programs improves treatment engagement. For each of the following statements, please rate the strength of your agreement with the statement from 1 (strongly disagree) to 5 (strongly agree).

IF EVIDENCE ASSESSMENT: BUPRENORPHINE FOR OPIOID USE DISORDER

1. In my opinion, ED-initiated buprenorphine in my ED with referral for ongoing MAT to community-based practices/programs will improve treatment engagement among patients with opioid use disorder.

Strongly Disagree	Disagree	Neither Agree nor Disagree	Agree	Strongly Agree	Don't Know	Not Applicable
1	2	3	4	5	98	99

2. Respected clinical experts in my institution feel that ED-initiated buprenorphine in my ED with referral for ongoing MAT to community-based practices/programs will improve treatment engagement among patients with opioid use disorder.

Strongly Disagree	Disagree	Neither Agree nor Disagree	Agree	Strongly Agree	Don't Know	Not Applicable
1	2	3	4	5	98	99

©2020 Hawk KF et al. *JAMA Network Open.*

3. Changes to improve and systematize ED-initiated buprenorphine in my ED with referral for ongoing MAT to community-based practices/programs to promote treatment engagement among patients with opioid use disorder:
    a. are supported by randomized controlled trials (RCTs) or other scientific evidence from my ED

Strongly Disagree	Disagree	Neither Agree nor Disagree	Agree	Strongly Agree	Don't Know	Not Applicable
1	2	3	4	5	98	99

    b. are supported by randomized controlled trials (RCTs) or other scientific evidence from other health care systems

Strongly Disagree	Disagree	Neither Agree nor Disagree	Agree	Strongly Agree	Don't Know	Not Applicable
1	2	3	4	5	98	99

    c. should be effective, based on current scientific knowledge

Strongly Disagree	Disagree	Neither Agree nor Disagree	Agree	Strongly Agree	Don't Know	Not Applicable
1	2	3	4	5	98	99

4. ED-initiated buprenorphine in my ED with referral for ongoing MAT to community-based practices/programs to promote treatment engagement among patients with an opioid use disorder:
    a. is supported by clinical experience with my ED's patients

Strongly Disagree	Disagree	Neither Agree nor Disagree	Agree	Strongly Agree	Don't Know	Not Applicable
1	2	3	4	5	98	99

b. is supported by clinical experiences with patients in other health care systems

Strongly Disagree	Disagree	Neither Agree nor Disagree	Agree	Strongly Agree	Don't Know	Not Applicable
1	2	3	4	5	98	99

c. conforms to the opinions of clinical experts in this setting

Strongly Disagree	Disagree	Neither Agree nor Disagree	Agree	Strongly Agree	Don't Know	Not Applicable
1	2	3	4	5	98	99

5. ED-initiated buprenorphine in my ED with referral for ongoing MAT to community-based practices/programs will improve health outcomes among patients with an opioid use disorder:
   a. has been well-accepted by my ED's patients in a pilot study

Strongly Disagree	Disagree	Neither Agree nor Disagree	Agree	Strongly Agree	Don't Know	Not Applicable
1	2	3	4	5	98	99

b. is consistent with clinical practices that have been accepted by my ED's patients

Strongly Disagree	Disagree	Neither Agree nor Disagree	Agree	Strongly Agree	Don't Know	Not Applicable
1	2	3	4	5	98	99

c. take into consideration the needs and preferences of my ED's patients

Strongly Disagree	Disagree	Neither Agree nor Disagree	Agree	Strongly Agree	Don't Know	Not Applicable
1	2	3	4	5	98	99

d. appear to have more advantages than disadvantages for my ED's patients

Strongly Disagree	Disagree	Neither Agree nor Disagree	Agree	Strongly Agree	Don't Know	Not Applicable
1	2	3	4	5	98	99

©2020 Hawk KF et al. *JAMA Network Open.*

II. CONTEXT ASSESSMENT

The following set of questions is about your experiences in your ED. For each of the following statements, please indicate how frequently you have observed the following sets of behaviors, from 1 (very infrequently) to 5 (very frequently).

1. How frequently have you observed senior leadership/clinical management (e.g. medical director) in your ED:
   a. reward clinical innovation and creativity to improve patient care

Very Infrequently	Infrequently	Neither Frequently nor Infrequently	Frequently	Very Frequently	Don't Know	Not Applicable
1	2	3	4	5	98	99

   b. solicit opinions of clinical staff regarding decisions about patient care

Very Infrequently	Infrequently	Neither Frequently nor Infrequently	Frequently	Very Frequently	Don't Know	Not Applicable
1	2	3	4	5	98	99

   c. seek ways to improve patient education and increase patient participation in treatment

Very Infrequently	Infrequently	Neither Frequently nor Infrequently	Frequently	Very Frequently	Don't Know	Not Applicable
1	2	3	4	5	98	99

©2020 Hawk KF et al. *JAMA Network Open.*

2. How frequently have you observed staff members in your ED:

a. have a sense of personal responsibility for improving patient care and outcomes

Very Infrequently	Infrequently	Neither Frequently nor Infrequently	Frequently	Very Frequently	Don’t Know	Not Applicable
1	2	3	4	5	98	99

b. cooperate to maintain and improve effectiveness of patient care

Very Infrequently	Infrequently	Neither Frequently nor Infrequently	Frequently	Very Frequently	Don’t Know	Not Applicable
1	2	3	4	5	98	99

c. be willing to innovate and/or experiment to improve clinical procedures

Very Infrequently	Infrequently	Neither Frequently nor Infrequently	Frequently	Very Frequently	Don’t Know	Not Applicable
1	2	3	4	5	98	99

d. be receptive to change in clinical processes

Very Infrequently	Infrequently	Neither Frequently nor Infrequently	Frequently	Very Frequently	Don’t Know	Not Applicable
1	2	3	4	5	98	99

©2020 Hawk KF et al. *JAMA Network Open.*

3. How frequently have you observed senior leadership/clinical management (e.g. medical director) in your ED:
   a. provide effective management for continuous improvement of patient care

Very Infrequently	Infrequently	Neither Frequently nor Infrequently	Frequently	Very Frequently	Don't Know	Not Applicable
1	2	3	4	5	98	99

   b. clearly define areas of responsibility and authority for clinical managers and staff

Very Infrequently	Infrequently	Neither Frequently nor Infrequently	Frequently	Very Frequently	Don't Know	Not Applicable
1	2	3	4	5	98	99

   c. promote team building to solve clinical care problems

Very Infrequently	Infrequently	Neither Frequently nor Infrequently	Frequently	Very Frequently	Don't Know	Not Applicable
1	2	3	4	5	98	99

   d. promote communication among clinical services and units

Very Infrequently	Infrequently	Neither Frequently nor Infrequently	Frequently	Very Frequently	Don't Know	Not Applicable
1	2	3	4	5	98	99

©2020 Hawk KF et al. *JAMA Network Open.*

4. How frequently have you observed senior leadership/clinical management (e.g. medical director) in your ED:

a. provide staff with information on your ED's performance measures and guidelines

Very Infrequently	Infrequently	Neither Frequently nor Infrequently	Frequently	Very Frequently	Don't Know	Not Applicable
1	2	3	4	5	98	99

b. establish clear goals for patient care processes and outcomes

Very Infrequently	Infrequently	Neither Frequently nor Infrequently	Frequently	Very Frequently	Don't Know	Not Applicable
1	2	3	4	5	98	99

c. provide staff members with feedback/data on effects of clinical decisions

Very Infrequently	Infrequently	Neither Frequently nor Infrequently	Frequently	Very Frequently	Don't Know	Not Applicable
1	2	3	4	5	98	99

d. hold staff members accountable for achieving results

Very Infrequently	Infrequently	Neither Frequently nor Infrequently	Frequently	Very Frequently	Don't Know	Not Applicable
1	2	3	4	5	98	99

©2020 Hawk KF et al. *JAMA Network Open.*

5. How frequently have you observed opinion leaders in your ED:

a. express belief that the current practice patterns can be improved

Very Infrequently	Infrequently	Neither Frequently nor Infrequently	Frequently	Very Frequently	Don't Know	Not Applicable
1	2	3	4	5	98	99

b. encourage and support changes in practice patterns to improve patient care

Very Infrequently	Infrequently	Neither Frequently nor Infrequently	Frequently	Very Frequently	Don't Know	Not Applicable
1	2	3	4	5	98	99

c. demonstrate willingness to try new clinical protocols

Very Infrequently	Infrequently	Neither Frequently nor Infrequently	Frequently	Very Frequently	Don't Know	Not Applicable
1	2	3	4	5	98	99

d. work cooperatively with senior leadership/clinical management (e.g. medical director) to make appropriate changes

Very Infrequently	Infrequently	Neither Frequently nor Infrequently	Frequently	Very Frequently	Don't Know	Not Applicable
1	2	3	4	5	98	99

©2020 Hawk KF et al. *JAMA Network Open.*

6. In general in your ED, when there is agreement that change needs to happen, how frequently have you or your colleagues:

a. had the necessary support in terms of budget or financial resources

Very Infrequently	Infrequently	Neither Frequently nor Infrequently	Frequently	Very Frequently	Don’t Know	Not Applicable
1	2	3	4	5	98	99

b. had the necessary support in terms of training

Very Infrequently	Infrequently	Neither Frequently nor Infrequently	Frequently	Very Frequently	Don’t Know	Not Applicable
1	2	3	4	5	98	99

c. had the necessary support in terms of facilities

Very Infrequently	Infrequently	Neither Frequently nor Infrequently	Frequently	Very Frequently	Don’t Know	Not Applicable
1	2	3	4	5	98	99

d. had the necessary support in terms of staffing

Very Infrequently	Infrequently	Neither Frequently nor Infrequently	Frequently	Very Frequently	Don’t Know	Not Applicable
1	2	3	4	5	98	99

©2020 Hawk KF et al. *JAMA Network Open.*
